# Clinical and radiological features of gastric and small intestinal anisakiasis: comparison with gastric ulcers and crohn’s disease

**DOI:** 10.1007/s11604-024-01731-z

**Published:** 2025-01-16

**Authors:** Kengo Ikejima, Daisuke Yamada, Natsuka Muraishi, Yasuyuki Kurihara

**Affiliations:** Department of Radiology, St. Luke’s International Hospital, 9-1 Akashi-cho, Chuo-ku, Tokyo, 104-8560 Japan

**Keywords:** Abdominal pain, Anisakiasis, Gastric ulcer, Crohn’s disease, Seafood

## Abstract

**Purpose:**

To compare the clinical and radiological features of gastric and small intestinal anisakiasis with those of gastric ulcers and Crohn’s disease.

**Materials and methods:**

In this retrospective cohort study, 205 cases of anisakiasis (148 gastric; 53 small intestinal) were identified between July 2003 and February 2022. The control groups included 130 and 31 patients with gastric ulcers and Crohn’s disease, respectively. Clinical and imaging findings were compared between the groups using the chi-square test, Fisher’s exact test, Mann–Whitney U test, and *t*-test.

**Results:**

Patients with gastric anisakiasis were younger (median age, 40 [21–85] years; 87 men) than those with gastric ulcers (median age, 64.5 [29–90] years; 101 men). Abdominal pain was common in the gastric anisakiasis group, whereas bleeding symptoms were frequent in the gastric ulcer group. Patients with small intestinal anisakiasis were older (mean age, 51.2 [38.6–63.7] years; 44 men) than those with Crohn’s disease (mean age, 35.9 [21.6–50.3] years; 22 men). Patients with gastric anisakiasis exhibited more edematous wall thickening, increased surrounding fat density, ascites, and thickening of other intestinal walls than those with gastric ulcers. Patients with small intestinal anisakiasis showed greater wall edema, perienteric fat stranding, proximal dilatation, clamp sign, and ascites than those with Crohn’s disease. Interobserver agreement was moderate to excellent, except for esophageal findings.

**Conclusion:**

Anisakiasis demonstrates clinical and radiological features distinct from those of gastric ulcers and Crohn’s disease. Recognizing these differences may aid in the differential diagnosis of gastrointestinal disorders, particularly in regions with high levels of raw fish consumption.

**Condensed abstract:**

This retrospective study compared CT findings of gastric and small intestinal anisakiasis with gastric ulcers and Crohn’s disease. Anisakiasis exhibited distinct features, including edematous wall thickening, increased surrounding fat density, and ascites. These findings can aid in differential diagnosis, particularly in regions where raw fish consumption is common.

## Introduction

Anisakiasis is a parasitic infection caused by the ingestion of seafood contaminated with third-stage larvae and commonly occurs through the consumption of raw or undercooked marine fish and cephalopods [1]. Although traditionally associated with countries that have dietary customs for consuming raw fish dishes, such as Japanese sashimi and sushi or Italian marinated crudo, the globalization of culinary practices and changes in health trends have led to frequent encounters with this disease worldwide. Anisakiasis causes symptoms via two main mechanisms: direct tissue damage and secondary allergic reactions [1, 2]. Patients with gastric anisakiasis typically experience acute, severe epigastric pain within hours of consuming contaminated fish. For intestinal anisakiasis, nonspecific clinical features such as nausea, vomiting, and diarrhea tend to appear slightly later [3]. The diagnostic challenge of anisakiasis lies in its similarity to the symptoms of gastric ulcers and Crohn’s disease, often leading to misdiagnosis if a detailed food consumption history is not obtained [4].

The parasite primarily infests the stomach and small intestine, with symptoms and radiological findings varying according to the location of the infestation. Gastric anisakiasis often presents with acute gastritis similar to that caused by gastric ulcers, whereas small intestinal anisakiasis may mimic enteritis, as seen in conditions such as Crohn’s disease, making the differential diagnosis challenging [5]. In the past, fluoroscopy was sometimes used to directly visualize the parasite [6, 7]. Currently, anisakiasis is commonly suspected based on patient history and CT findings. While gastric anisakiasis can be definitively diagnosed through endoscopic visualization and removal of the parasite, the diagnosis of intestinal anisakiasis relies more heavily on the combination of clinical and imaging findings with a history of raw fish consumption. Although specific IgE antibody testing is available, its clinical utility may be limited by the time required for results and potential cross-reactivity with other parasites.

Despite the existence of studies investigating the imaging features of gastric and small intestinal anisakiasis [3, 8, 9], comparative studies with other gastrointestinal disorders are still needed. While Takabayashi et al. demonstrated the differences in clinical manifestations, time courses, and CT findings between gastric and small intestinal anisakiasis in a large-scale study [3], and the clinical features of anisakiasis are well documented [1, 2], no systematic analysis has compared both the clinical and radiological features of anisakiasis with those of diseases that can mimic anisakiasis, such as gastric ulcers and Crohn’s disease. In this study, we expanded the patient cohort over an extensive period of approximately 20 years and used control groups to compare both the clinical and radiological features of gastric and small intestinal anisakiasis with those of gastric ulcers and Crohn’s disease, which may help improve the differential diagnosis of these conditions.

## Materials and methods

### Patient selection

This retrospective cohort study was approved by the institutional review board of our tertiary hospital. The requirement for informed consent was waived because of the retrospective nature of the study (research number: 23-R001).

For the anisakiasis group, using electronic medical records, we initially extracted 285 examinations from 273 patients (10 patients with two examinations and one patient with three examinations) who were registered in medical records with diagnoses of “anisakiasis,” “gastric anisakiasis,” “gastrointestinal anisakiasis,” or “intestinal anisakiasis” and underwent CT imaging of the abdomen within 7 days before or after diagnosis registration. Among these, 205 examinations from 195 patients (eight patients with two examinations and one patient with three examinations) were confirmed either by direct visualization of *Anisakis* larvae via endoscopy or by testing positive for *Anisakis* antibodies or *Anisakis*-specific IgE. Cases were classified as gastric anisakiasis when confirmed by endoscopy, while those positive for *Anisakis* antibodies or *Anisakis*-specific IgE were classified as either gastric or small intestinal anisakiasis based on medical record review. This yielded 152 gastric anisakiasis examinations (from 143 patients: seven patients with two examinations and one patient with three examinations) and 53 small intestinal anisakiasis examinations (from 52 patients: one patient with two examinations). After excluding 4 asymptomatic cases that were incidentally discovered during endoscopy and showed minimal imaging findings, 148 gastric anisakiasis examinations (from 139 patients: seven patients with two examinations and one patient with three examinations) and 53 small intestinal anisakiasis examinations (from 52 patients: one patient with two examinations) remained.

For the gastric ulcer control group, using the same method, we initially extracted 215 examinations from 214 patients (one patient with two examinations) who were registered in medical records with “gastric ulcer,” tested positive for *Helicobacter pylori*, and underwent CT imaging within 7 days before or after diagnosis registration. We excluded cases with different final diagnoses (36 examinations), those where the abdomen was not included in the CT scan range (2 examinations), and those without endoscopic confirmation of gastric ulcer within 2 weeks before or after diagnosis registration (47 examinations). This resulted in 130 examinations from 130 patients, all of whom were symptomatic.

For the Crohn’s disease control group, similarly, we initially extracted 51 patients registered in medical records with “Crohn’s disease” who underwent abdominal CT imaging within 7 days before or after diagnosis registration. After excluding asymptomatic and post-surgical cases, 35 patients remained. Further excluding cases without clinical diagnosis of small bowel inflammation resulted in 27 patients. Although these patients were initially registered with Crohn’s disease only once, three patients underwent multiple CT examinations during subsequent symptomatic episodes (two patients with two examinations and one patient with three examinations), resulting in 31 examinations from 27 patients.

### Image acquisition

Owing to the extended duration of the study, multiple CT scanners were used, including scanners manufactured by Canon Medical Systems Corporation (Aquilion ONE and Aquilion64) and GE Healthcare (HiSpeed NX/i, BrightSpeed Elite, LightSpeed RT16, Revolution CT, Optima CT660 Pro, and Revolution Maxima CT).

Unenhanced CT data were available for 64 examinations: 50 for gastric anisakiasis, 12 for gastric ulcers, 1 for small intestinal anisakiasis, and 1 for Crohn’s disease. Contrast-enhanced CT data were available for 301 examinations: 98 for gastric anisakiasis, 118 for gastric ulcers, 52 for small intestinal anisakiasis, and 30 for Crohn’s disease.

Contrast-enhanced images were obtained 90 s after intravenous administration of contrast media (80–135 mL of 300–350 mg/mL non-ionic contrast media, depending on the patient’s body weight). Reconstructed images with a 1.25–5 mm slice thickness were used for assessment.

### Image analysis

Based on the radiological findings of previous studies, case reports [6–8, 10–14], and the clinical experience of our institution, the following radiological features were assessed:

Gastric lesions: Edematous thickening of the stomach wall, localized protrusion of the gastric wall, increased perigastric fat density, ascites, edematous changes in other bowel segments (small intestine and colon), fluid collection around the esophagus, and esophageal wall thickening.

Small intestinal lesions: Small intestine wall thickening, submucosal edema (target sign) in the small intestine, increased density of surrounding fat tissue, intestinal dilatation, clamp sign (progressive thickening of intestinal wall at the site of caliber change), and ascites.

The intestines were classified as “dilated” when the bowel lumen was > 3 cm, measured from one outer wall to the opposite outer wall. The clamp sign was defined as a gradual thickening of the intestinal wall at the transition point between dilated and non-dilated bowel segments, as described by Chen et al. [14]. Two radiologists with six (K.I.) and nine (D.Y.) years of experience independently reviewed the CT images. In cases of differing assessments, an abdominal radiologist with 18 years of experience (N.M.) reviewed the images to reach a consensus.

### Clinical information

For all 362 cases, we collected the following data from medical records: demographic information (age and sex); clinical symptoms (abdominal pain, hematochezia, loss of appetite, hematemesis, fever, rash, nausea or vomiting, and altered mental status); laboratory findings (results of white blood cell count [WBC], C-reactive protein levels [CRP], and blood eosinophil cell count [EOS]).

### Statistical analysis

Given the retrospective nature of this study, a formal power calculation was not performed. Patients with unavailable data were excluded from the analysis. Data are presented as the mean ± standard deviation or median (range) for continuous variables, such as age and Ki-67, and as numbers (percentages) for categorical variables. We used the *t*-test and Mann–Whitney U test for continuous variables and the chi-square and Fisher’s exact tests for categorical variables. Continuous variables were verified by Shapiro–Wilk statistics. A *p*-value < 0.05 was considered statistically significant.

Interobserver agreement was assessed by using κ values and was interpreted as poor (κ < 0.20), fair (κ = 0.21–0.40), moderate (κ = 0.41–0.60), good (κ = 0.61–0.81), or excellent (κ = 0.81–1.00). All statistical analyses were performed (K.I.) using EZR4.3.1 (Saitama Medical Center, Jichi Medical University, Saitama, Japan), which is a graphical user interface for R (The R Foundation for Statistical Computing, Vienna, Austria) [15].

## Results

**Fig. 1:**
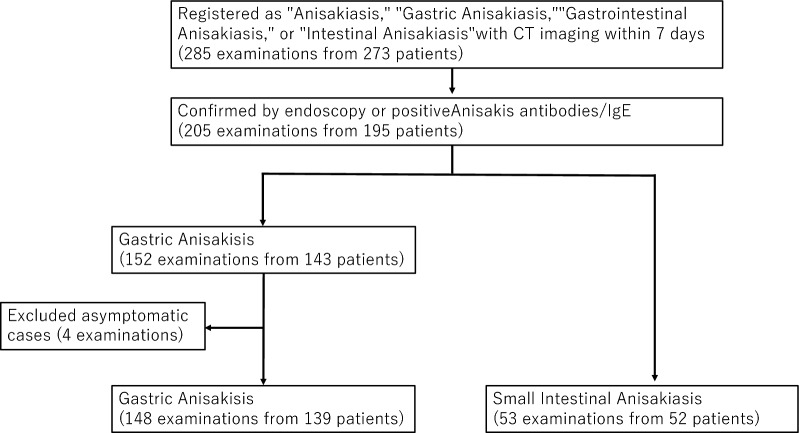
Flowchart of patient selection of anisakiasis cases

**Fig. 2:**
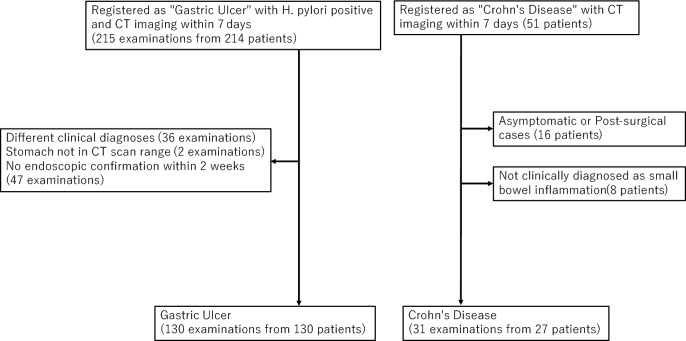
Flowchart of patient selection of comparison cases

Our study included 148 gastric anisakiasis examinations (from 139 patients) and 53 small intestinal anisakiasis examinations (from 52 patients). The control groups consisted of 130 gastric ulcer examinations (from 130 patients) and 31 Crohn’s disease examinations (from 27 patients). The patient selection process is shown in Figs. 1 and 2.

### Clinical findings

Table 1 summarizes the clinicopathological characteristics of the gastric cases. In the gastric group, continuous variables such as age and EOS were analyzed using the Mann–Whitney U test. The median age was 40 (range, 21–85) years for patients with gastric anisakiasis and 64.5 (range, 29–90) years for patients with gastric ulcers, with a significant age difference between the groups (*p* < 0.001). The EOS levels were significantly higher in patients with gastric anisakiasis (*p* = 0.02) than in those with gastric ulcers. Patients with gastric anisakiasis predominantly experienced abdominal pain (*p* < 0.001), and bleeding symptoms were rare, with only two cases of hematochezia and one case of hematemesis. In contrast, patients with gastric ulcers had an increased incidence of bleeding symptoms, such as hematochezia (*p* < 0.001) and hematemesis (*p* < 0.001).

**Table 1:** Clinicopathological characteristics of gastric cases

Variable	Gastric anisakiasis (n = 148)	Gastric ulcer (n = 130)	*p*-value
Patient age (y), median (range)	40 (21–85)	64.5 (29–90)	< 0.001
Sex			
Male	87 (59)	101 (78)	< 0.001
Female	61 (41)	29 (22)	
WBC (10^3^/μL), median (range)	10.2 (4.8–27.2)	11.2 (2.5–35.6)	0.116
CRP (mg/dL), median (range)	0.79 (0.04–11.92)	0.21 (0.04–17.58)	0.186
EOS (%), median (range)	1.85 (0.2–8.5)	1 (0.2–23.5)	0.02
Symptoms			
Abdominal pain	143 (97)	50 (38)	< 0.001
Hematochezia	2 (1)	66 (51)	< 0.001
Loss of appetite	0 (0)	1 (1)	0.468
Hematemesis	1 (1)	50 (38)	< 0.001
Fever	1 (1)	0 (0)	1
Rash	5 (3)	0 (0)	0.063
Nausea or vomiting	15 (10)	18 (14)	0.359
Altered mental status	0 (0)	10 (8)	< 0.001

Continuous variables are presented as median (range) or mean ± standard deviation, and categorical variables are presented as number (percentage), unless otherwise indicated

*CRP* C-reactive protein; *EOS* blood eosinophilic cell count; *WBC* white blood cell;

Table 2 summarizes the clinicopathological characteristics of intestinal cases. For the small bowel group, age was normally distributed and analyzed using the *t*-test, whereas other continuous variables, such as EOS, were analyzed using the Mann–Whitney U test. The mean age was 51.2 (38.6–63.7) years for patients with small bowel anisakiasis and 35.9 (standard deviation, 21.6–50.3) years for patients with Crohn’s disease, with a significant age difference between the groups (*p* < 0.001). The EOS levels were significantly higher in patients with small bowel anisakiasis (*p* < 0.001) than in those with Crohn’s disease. Hematochezia was significantly more common in patients with Crohn’s disease (*p* = 0.0163) than in those with small bowel anisakiasis. Nausea or vomiting was significantly more common in patients with small bowel anisakiasis (*p* = 0.0483) than in those with Crohn’s disease. The prevalence of other symptoms did not differ significantly between the two groups.

**Table 2:** Clinicopathological characteristics of intestinal cases

Variable	Small bowel anisakis (n = 53)	Crohn’s disease (n = 31)	*p*
Patient age (y), mean (standard deviation)	51.2 (38.6–63.7)	35.9 (21.6–50.3)	< 0.001
Sex			
Male	44 (83)	22 (71)	0.271
Female	9 (17)	9 (29)	
WBC, median (range)	10.45 (4.3–18.4)	11.7 (5.1–26.1)	0.231
CRP, median (range)	3.555 (0.05–13.77)	3.49 (0.09–19.82)	0.561
EOS, median (range)	4.65 (0–20.7)	1.5 (0.5–8.1)	< 0.001
Symptoms			
Abdominal pain	49 (92)	26 (84)	0.28
Hematochezia	0 (0)	4 (13)	0.0163
Hematemesis	0 (0)	1 (3)	0.37
Fever	4 (8)	3 (10)	0.705
Rash	1 (2)	0 (0)	1
Nausea or vomiting	20 (38)	5 (16)	0.0483
Altered mental status	0 (0)	1 (3)	0.369

Continuous variables are presented as median (range) or mean ± standard deviation, and categorical variables are presented as number (percentage), unless otherwise indicated

*CRP* C-reactive protein; *EOS* blood eosinophilic cell count; *WBC* white blood cell;

### Imaging findings

For gastric cases, interobserver agreement was excellent for wall thickening (κ = 0.796), good for localized protrusion of gastric wall (κ = 0.691) and increased density of perigastric fat (κ = 0.663), and moderate for edematous changes in other bowel segments (κ = 0.527) and ascites (κ = 0.482). However, fluid collection around the esophagus (κ = 0) and esophageal wall thickening (κ = – 0.006) showed poor interobserver agreement. For small intestinal cases, interobserver agreement was good for clamp sign (κ = 0.603) and moderate for all other findings: increased density of surrounding fat tissue (κ = 0.588), ascites (κ = 0.509), intestinal dilatation (κ = 0.504), and submucosal edema (target sign) (κ = 0.464).

**Fig. 3:**
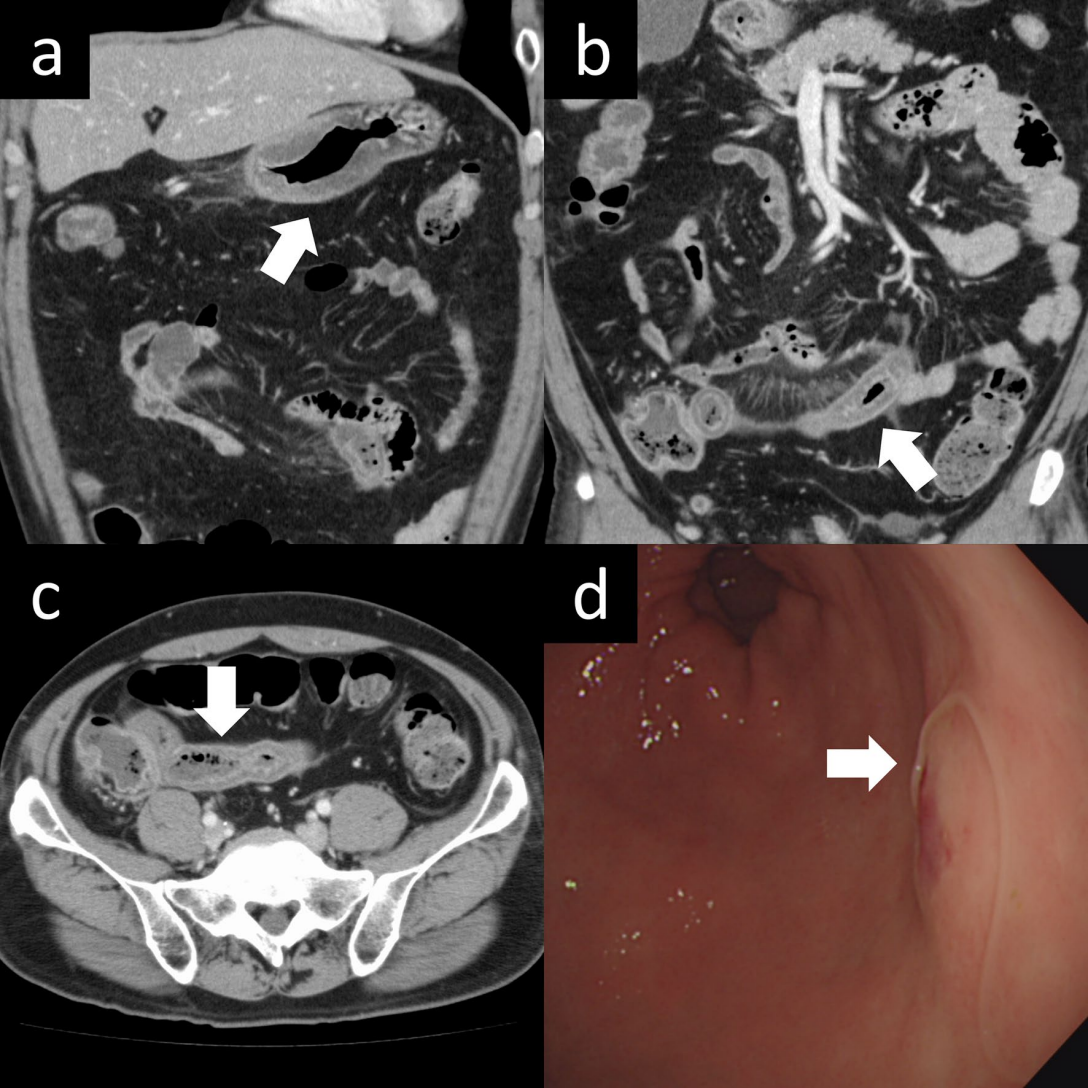
A 55-year-old man presented to the emergency department with a complaint of abdominal pain since the day before. Contrast-enhanced CT images in the coronal plane demonstrate edematous wall thickening of the stomach and increased density of the surrounding fat tissue (Fig. 3a, arrow). Similar edematous wall thickening and increased fat density are seen in the small intestine and colon at the ileocecal region on coronal and axial contrast-enhanced CT images (Fig. 3b, C, arrows). An *Anisakis* larva was identified and removed during upper gastrointestinal endoscopy (Fig. 3d, arrow)

**Fig. 4:**
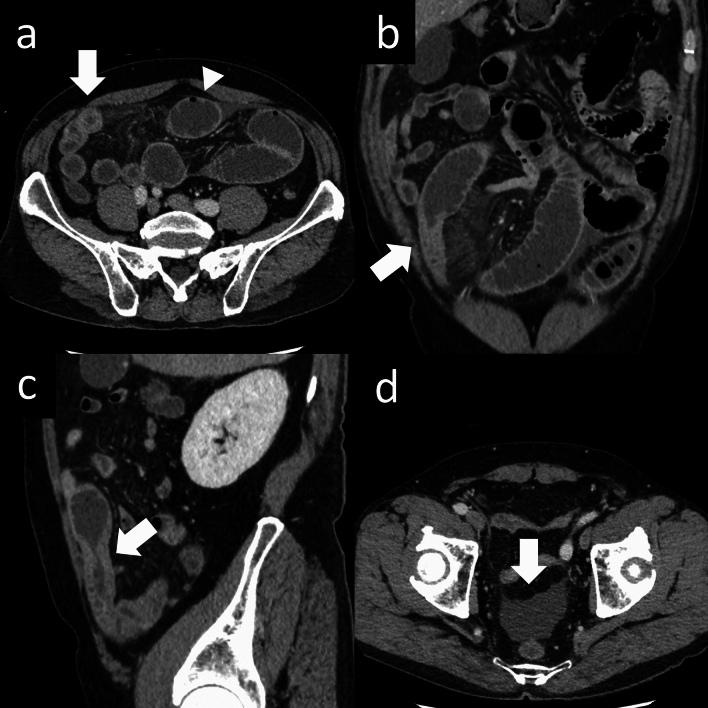
A 56-year-old man presented to the emergency department with a complaint of abdominal pain since the previous day. Axial post-contrast CT images show edematous wall thickening of the small intestine (Fig. 4a, arrow), along with proximal small bowel dilatation (Fig. 4a, arrowhead) and increased fat density. Coronal and sagittal post-contrast CT images demonstrate progressive intestinal wall thickening at the site of caliber change (clamp sign, Fig. 4b, c, arrows). Ascites is noted in the pelvic cavity (Fig. 4d, arrow)

Table 3 summarizes the imaging findings of gastric cases. In patients with stomach anisakiasis, edematous wall thickening, increased surrounding fat density, ascites, and thickening of other intestinal walls were significantly more common than in those with gastric ulcers (Fig. 3). Gastric wall protrusions were frequently observed in patients with gastric ulcers. Table 4 summarizes the imaging findings of intestinal cases. Patients with small intestinal anisakiasis exhibited significantly more instances of edematous wall thickening, increased surrounding fat density, proximal intestinal dilation, clamp sign, and ascites than those with Crohn’s disease (Fig. 4).

**Table 3:** Imaging findings of gastric cases

Variable	Gastric anisakis (n = 148)	Gastric ulcer (n = 130)	*p*	κ
Wall thickening	141 (95)	27 (21)	< 0.001	0.796
Localized protrusion of gastric wall	1 (1)	43 (33)	< 0.001	0.691
Increased density of perigastric fat	127 (86)	28 (22)	< 0.001	0.663
Ascites	59 (40)	7 (5)	< 0.001	0.482
Edematous changes in other bowel segments	12 (8)	1 (1)	0.004	0.527
Fluid collection around the esophagus	4 (3)	0 (0)	0.125	0.000
Esophageal wall thickening	5 (3)	1 (1)	0.22	− 0.006

Categorical variables are presented as number (percentage)

Kappa indicates interobserver reliability of two raters

**Table 4:** Imaging findings of intestinal cases

Variable	Small bowel anisakis (n = 53)	Crohn’s disease (n = 31)	*p*	κ
Submucosal edema (target sign)	46 (87)	6 (19)	< 0.001	0.464
Increased density of surrounding fat tissue	48 (91)	12 (39)	< 0.001	0.588
intestinal dilatation (> 3 cm)	42 (79)	11 (35)	< 0.001	0.504
Clamp sign	39 (74)	6 (19)	< 0.001	0.603
Ascites	49 (92)	14 (45)	< 0.001	0.509

Categorical variables are presented as number (percentage)

Kappa indicates interobserver reliability of two raters

## Discussion

We retrospectively compared the radiological features of gastric and small intestinal anisakiasis with those of gastric ulcers and Crohn’s disease. Gastric and small intestinal anisakiasis cases exhibited distinct clinical and radiological features, with patients having gastric anisakiasis being younger, more frequently presenting with abdominal pain, and demonstrating edematous wall thickening, increased surrounding fat density, ascites, and thickening of other intestinal walls. Patients with small intestinal anisakiasis were older and exhibited greater wall edema, perienteric fat stranding, proximal dilatation, clamp sign and ascites compared to those with Crohn’s disease. Based on these observations, our study presents several novel contributions to the field of anisakiasis imaging. Through this comprehensive comparison of radiological features between anisakiasis and control groups, we have identified distinct imaging characteristics that can aid in differential diagnosis. Specifically, our study is the first to systematically evaluate the clamp sign in small intestinal anisakiasis and provide data on multiple segment involvement in a Japanese population.

Patients in the gastric anisakiasis group were younger than those in the gastric ulcer group. Older patients in Japan often remain untreated for *H. pylori* infection, which contributes to the prevalence of gastric ulcers [16]. The elevated EOS levels suggest the contribution of allergic reactions to anisakiasis. Abdominal pain symptoms were common in gastric anisakiasis, whereas bleeding symptoms were infrequent, possibly because gastric ulcers are chronic and may present with intermittent bleeding.

Radiologically, gastric anisakiasis was characterized by gastric wall edema, ascites, increased perigastric fat density, and edematous changes in other bowel segments, suggesting the role of tissue damage and allergic reactions. The simultaneous involvement of multiple intestinal segments was observed in our cohort, although less frequently than in other recent studies. Chen Zhou et al. [14] reported multiple segment involvement in 34% of their cases, particularly noting concurrent involvement of the gastric antrum and small intestinal segments. Similarly, Fornell-Perez et al. [9] reported a higher incidence of multiple lesions compared to our findings. These variations in the frequency of multiple segment involvement might be attributed to differences in healthcare accessibility and imaging practices rather than regional factors, as our institution’s location in an urban area of Japan enables early medical consultation and prompt CT imaging. This early detection might lead to diagnosis and treatment before the development of multiple lesions or parasite migration.

Gastric ulcers present with epigastric discomfort, nausea, and abdominal pain. Although endoscopy is the diagnostic standard for anisakiasis and gastric ulcers, CT is often performed when larvae cannot be visually confirmed [17]. Both conditions can exhibit edematous wall thickening; however, wall protrusions suggest gastric ulcers, whereas ascites supports anisakiasis.

Patients with small bowel anisakiasis were older than those with Crohn’s disease, which typically affects younger individuals. Increased EOS were noted, suggesting that an allergic reaction to anisakiasis may have contributed to eosinophilia. While most symptoms were not significantly different, four cases of enteritis had hematochezia, possibly due to ulcerative lesions commonly seen in Crohn’s disease, leading to bleeding.

Small bowel anisakiasis displayed radiological findings of wall edema, perienteric fat stranding, proximal bowel dilatation with characteristic clamp sign and ascites more frequently than Crohn’s disease. Similar to gastric anisakiasis, these symptoms may result from allergic reactions to larval infestations. Small intestinal anisakiasis is often misdiagnosed as another small bowel disease, such as Crohn’s disease [5]. Upstream bowel dilatation was particularly prevalent, often resembling the imaging findings of common small bowel obstruction. However, edematous wall thickening at the obstruction site can help differentiate anisakiasis from other causes. This suggests that inflammation or direct larval invasion impairs intestinal motility, leading to obstruction. At the site of caliber change, we evaluated the presence of clamp sign, defined as progressive thickening of the intestinal wall, which was recently described by Chen Zhou et al. [14]. This finding was observed in almost all cases of small intestinal anisakiasis and was significantly more frequent than in Crohn’s disease. The clamp sign reflects the pathophysiology of anisakiasis, where Anisakis larvae trigger both direct tissue damage and allergic reactions [1, 2]. This inflammatory process results in gradual thickening of the intestinal wall, creating a characteristic clamp-like appearance at the transition point. This gradual transition particularly helps differentiate anisakiasis from strangulated bowel obstruction, which typically shows short, abrupt narrowing (beak sign or fat notch sign) at the point of obstruction [18]. While both conditions can present with bowel wall edema and obstruction, the presence of the clamp sign versus an abrupt transition point serves as a crucial differentiating feature, which is particularly valuable in cases where other imaging findings may overlap. However, the clamp sign was not exclusively specific to anisakiasis, as it was also observed in some cases of Crohn’s disease. We hypothesize that active inflammation in Crohn’s disease may cause bowel obstruction with progressive wall thickening similar to the clamp sign, while chronic adhesions in Crohn’s disease may result in more abrupt caliber changes typically seen in mechanical obstruction.

Our study builds on previous studies by including additional cases and performing comparative analyses [3, 8]. The congruence of our results with those of previous studies corroborates the presence of edematous changes and ascites as common findings in anisakiasis. By analyzing a large cohort with control groups, our study makes several novel contributions. First, we provide the first data on the prevalence of multiple segment involvement in a Japanese population. Second, our systematic evaluation of the clamp sign at sites of caliber change adds to the radiological features that can help differentiate small intestinal anisakiasis from other gastrointestinal disorders. These findings, derived from comparison with control groups, enhance our understanding of the characteristic imaging features of anisakiasis and may improve its diagnostic accuracy in clinical practice.

Our study has several limitations. First, its retrospective design precluded a formal power calculation, although we believe our sample size was sufficient to detect clinically meaningful differences in imaging findings. Second, the single-center nature and extended study period, which encompassed varying CT imaging technologies, slice thicknesses, and contrast use, complicated standardization with control groups. Advancements in CT technology over this 20-year period may have influenced image quality through improved spatial resolution and reconstruction algorithms. However, the key imaging features we evaluated are relatively robust findings that should be reliably detectable across different generations of CT scanners. To minimize potential variability, we established clear, objective criteria for each imaging finding, such as using specific measurement thresholds for bowel dilatation. Furthermore, the clamp sign represents a morphological pattern that depends more on recognition of the overall bowel configuration rather than fine detail resolution. Third, anisakiasis findings in the esophagus were limited by the small number of cases and low inter-reader agreement. These factors may limit the generalizability of our results to other populations and settings. Nevertheless, the inclusion of control groups in our study enhances the comparative value of our findings.

In conclusion, patients with gastric anisakiasis were younger, more frequently presented with abdominal pain, and demonstrated edematous wall thickening, increased surrounding fat density, and ascites on CT when compared to patients with gastric ulcers. Notably, although rare, thickening of other intestinal walls was observed in gastric anisakiasis. Patients with small intestinal anisakiasis were older and exhibited greater wall edema, perienteric fat stranding, ascites, and, importantly, proximal bowel dilatation with characteristic clamp sign at the site of caliber change when compared to patients with Crohn’s disease. These findings can aid in the differential diagnosis of anisakiasis and other gastrointestinal disorders. Future prospective studies with larger patient populations and standardized imaging protocols could validate these findings and further explore the unique radiological characteristics of anisakiasis.
